# Supplementary material for: Exploring ways to support patients with noncommunicable diseases: A pilot study in Nepal during the COVID-19 pandemic
Source: PLOS Glob Public Health. 2024 Jul 19;4(7):e0003509. doi: 10.1371/journal.pgph.0003509 (PMC11259295; doi:10.1371/journal.pgph.0003509)
Supplement: S1 Text — (DOCX) [file pgph.0003509.s001.docx]

**S1 Text. Interview guide**

**For patients with type 2 diabetes**

**Name of participant:**

**Age:**

**Address:**

**Contact phone no.:**

**Date of interview:**

1. How long have you been diagnosed as a patient with NCD (CVD or diabetes)? How important is it for you to visit for consultation? (***Probe items:*** duration, changes in drugs, quantity, complications frequency, understanding about disease process, consultation on co-morbidities)
2. What are your experiences in seeking and receiving health services before and during the COVID-19 pandemic?
3. How has Covid-19 affected your consultations and follow-ups?
4. Please tell us the difficulties that you are facing in terms of access to and utilization of health services during this COVID-19 pandemic.

***(Probe items:*** fear related to COVID, availability of health care providers and medicines, difficulty in getting transport services, etc.)

1. Please tell us the best ways to address these problems to gain access to and use NCD-related services effectively and easily.

***Probe items:***

1. Telemedicine: Consultation with doctors and nurses through the Internet or by phone
2. Medications: stock availability
3. Provision of free government services
4. Insurance facilities

6. Please tell us any other suggestion you may have.

**For healthcare providers**

**Name of participant:**

**Age:**

**Position:**

**Health facility:**

**Work experience: ……….. Years**

**Work duration at the facility:**

**Contact phone no.:**

**Date of interview:**

**A. Noncommunicable diseases (NCDs) and related services:**

1. In normal context, or say before the COVID-19 pandemic started, what type of NCD and related services were being implemented from this health facility/hospital?

(***Probe items:*** NCD such as diabetes screening, lab test, consultation, prescription, etc.)

1. What is different now than before in terms of delivering NCD-related services from your health facility?
2. Please tell us about the disruption of delivering health services during the COVID-19 pandemic.

(***Probe items:*** delivery of overall general health services—delivery of NCD-related health services and specific NCD services being disrupted)

1. What are the major causes of disruption in the NCD services delivery?

(***Probe items:*** Travel restriction, adequacy of the number of human resources/staff, supply logistics, medication, and others)

1. What are the best strategies/ways to address the problem of disruption in NCD services delivery?

(***Probe items:*** Use of telemedicine, novel ways of supply chain management, use of other community-level healthcare workers/volunteers to provide NCD medication/services at the doorsteps of patients, and other suggestions)

1. Any other suggestions you may have in terms of NCD services delivery during the COVID-19 pandemic and future similar pandemic situation would be appreciated.
   1. …………………………………………………..
   2. …………………………………………………..
   3. ………………………………………………….
