# Supplementary material for: Exploring ways to support patients with noncommunicable diseases: A pilot study in Nepal during the COVID-19 pandemic
Source: PLOS Glob Public Health. 2024 Jul 19;4(7):e0003509. doi: 10.1371/journal.pgph.0003509 (PMC11259295; doi:10.1371/journal.pgph.0003509)
Supplement: S2 Text — (DOCX) [file pgph.0003509.s002.docx]

**S2 Text. Data analysis**

1. **Multiple correspondence analysis (MCA) and Hierarchical Cluster Analysis (HCA)**

An MCA was conducted to determine the interrelationships among categorical variables through visualization using the FactoMineR package [23]. Most associated categories were plotted close together on the calculated values. Unassociated ones were plotted far apart. Our questionnaire data contained 31 variables, all of which were included in the analysis. To improve the readability of the graph, only the most relevant variables were included in the plots in some cases, even for analyses that were based on the total data. HCA was used to divide a population into subgroups, through a distance matrix, to determine the main clusters revealed by the MCA analysis.

1. **Classification tree analysis (CTA)**

We performed CTA to identify variables or combinations of variables that enhance the relevant characteristics, using survey data. The CTA was based on the Classification and Regression Trees algorithm using the rpart R package [25]. Contributing factors to the characteristics of patients with NCDs were analyzed by CTA, using variables generated from the choices listed in the questionnaire. Based on the exploratory method, the pattern was identified as notable results, which included only variables that contributed significantly to sub-typing patients in the classification tree. A p-value<0.05 was considered statistically significant. The 95% confidence interval (CI) for the proportion of objective variables in each subgroup was calculated using the Clopper–Pearson method. Using CTA, we extracted the pattern for various combinations of questions and the important contributing factors to characterize the patients. This process made the results easily understandable with simple explanation.
