# Supplementary figures and images for: Exploring ways to support patients with noncommunicable diseases: A pilot study in Nepal during the COVID-19 pandemic
Source: PLOS Glob Public Health. 2024 Jul 19;4(7):e0003509. doi: 10.1371/journal.pgph.0003509 (PMC11259295; doi:10.1371/journal.pgph.0003509)

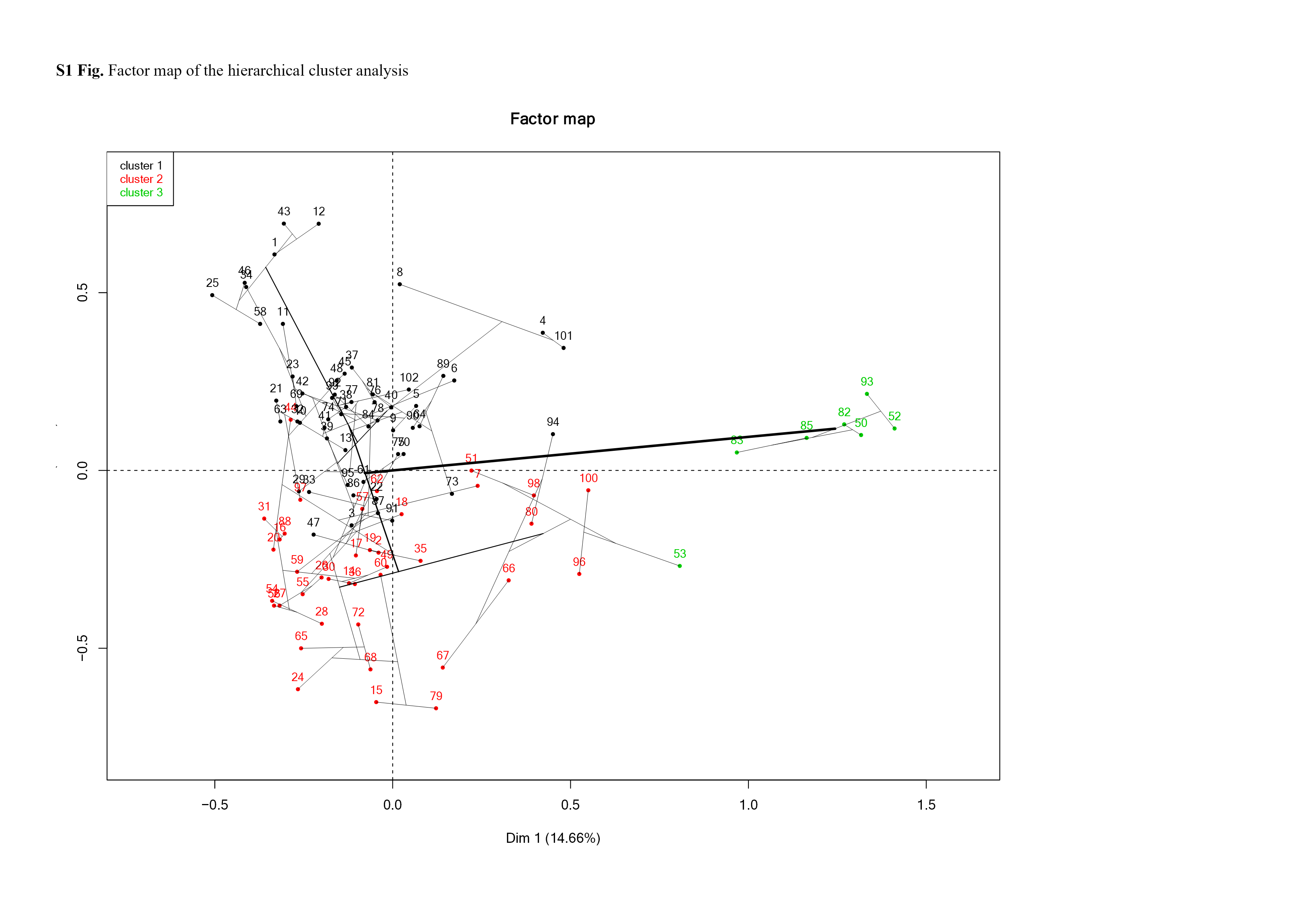

Supplement: S1 Fig — (TIFF) [file pgph.0003509.s003.tiff]
