# Supplementary material for: Exploring ways to support patients with noncommunicable diseases: A pilot study in Nepal during the COVID-19 pandemic
Source: PLOS Glob Public Health. 2024 Jul 19;4(7):e0003509. doi: 10.1371/journal.pgph.0003509 (PMC11259295; doi:10.1371/journal.pgph.0003509)
Supplement: S1 Table — (DOCX) [file pgph.0003509.s004.docx]

**S1 Table.** Differences in responses in each cluster

| Categories | Variables | Abbreviation_2 | Answers | Cluster 1 | | Cluster 2 | | Cluster 3 | | Total | |
| --- | --- | --- | --- | --- | --- | --- | --- | --- | --- | --- | --- |
| Self-perceived difficulty in managing NCDs | Self-perceived difficulty in obtaining patients’ medicines | dm_y | Yes | 9 | 16% | **22** | **56%** | 0 | 0% | 31 | 30% |
|  |  | dm_n | No | 47 | 84% | 17 | 44% | 7 | 100% | 71 | 70% |
|  | Self-perceived difficulty in accessing routine medical care | dc_y | Yes | 20 | 36% | 35 | 90% | 5 | 71% | 60 | 59% |
|  |  | dc_n | No | **36** | **64%** | 4 | 10% | 2 | 29% | 42 | 41% |
| Psychological impact caused by the COVID-19 pandemic | Concerns about COVID-19 | co_y | Yes | 19 | 34% | 37 | 95% | 4 | 57% | 60 | 59% |
|  |  | co_nt | No | **37** | **66%** | 2 | 5% | 3 | 43% | 42 | 41% |
|  | Nervousness caused by news on COVID-19 on social media | ne_a | Agree | 6 | 11% | **31** | **79%** | 1 | 14% | 38 | 37% |
|  |  | ne_n | No | 50 | 89% | 8 | 21% | 6 | 86% | 64 | 63% |
| Lifestyle behavior changes | Frequency of communication | fr_l | not less | 23 | 41% | 9 | 23% | **6** | **86%** | 38 | 37% |
|  |  | fr_n | less | 33 | 59% | 30 | 77% | 1 | 14% | 64 | 63% |
|  | Sleep problems | sl_n | Yes | 2 | 4% | 13 | 33% | 1 | 14% | 16 | 16% |
|  |  | sl_y | No | 54 | 96% | 26 | 67% | 6 | 86% | 86 | 84% |
|  | Smoking | sm_y | Yes | 10 | 18% | 2 | 5% | 1 | 14% | 13 | 13% |
|  |  | sm_n | No | 46 | 82% | 37 | 95% | 6 | 86% | 89 | 87% |
|  | Alcohol consumption | dr_y | Yes | 9 | 16% | 1 | 3% | 0 | 0 | 10 | 10% |
|  |  | dr_n | No | 47 | 84% | 38 | 97% | 7 | 100% | 92 | 90% |
|  | Physical activity | ph_y | Yes | 30 | 54% | 20 | 51% | 7 | 100% | 57 | 56% |
|  |  | ph_n | No | 26 | 46% | 19 | 49% | 0 | 0% | 45 | 44% |
| Precautionary behaviors | Wearing a face mask | Fa_y | Yes | 47 | 84% | 38 | 97% | 7 | 100% | 92 | 90% |
|  |  | Fa_n | No | 9 | 16% | 1 | 3% | 0 | 0% | 10 | 10% |
|  | Washing/Sanitizing one’s hands | Sa_y | Yes | 47 | 84% | 38 | 97% | 7 | 100% | 92 | 90% |
|  |  | Sa_n | No | 9 | 16% | 1 | 3% | 0 | 0% | 10 | 10% |
|  | Avoiding public places/crowds | Ac_y | Yes | 47 | 84% | 34 | 87% | 7 | % | 88 | % |
|  |  | Ac_n | No | 9 | 16% | 5 | 13% | 0 | % | 14 | % |
|  | Avoiding in-person contact with high-risk people | Ap_y | Yes | 8 | 14% | 13 | 33% | **7** | **100%** | 28 | 27% |
|  |  | Ap_n | No | 48 | 86% | 26 | 67% | 0 | 0% | 74 | 73% |
|  | Cancelling a doctor’s appointment | Cd_y | Yes | 6 | 11% | 8 | 21% | **7** | **100%** | 21 | 21% |
|  |  | Cd_n | No | 50 | 89% | 31 | 79% | 0 | 0% | 81 | 79% |
|  | Avoiding in-person contact with friends or family | Af_y | Yes | 4 | 7% | 8 | 21% | **7** | **100%** | 19 | 19% |
|  |  | Af_n | No | 51 | 93% | 31 | 79% | 0 | 0% | 83 | 81% |
|  | Cancelling/postponing travel | CT_y | Yes | 4 | 7% | 4 | 10% | **5** | **71%** | 13 | 13% |
|  |  | CT_n | No | 52 | 93% | 35 | 90% | 2 | 29% | 89 | 87% |
|  | Stockpiling food/water | St_y | Yes | 3 | 5% | 5 | 13% | **4** | **57%** | 12 | 12% |
|  |  | St_n | No | 53 | 95% | 34 | 87% | 3 | 43% | 90 | 88% |
|  | Praying | Pr_y | Yes | 4 | 7% | 1 | 3% | **5** | **71%** | 10 | 10% |
|  |  | Pr_n | No | 52 | 93% | 38 | 97% | 2 | 29% | 92 | 90% |
|  | Visiting a doctor in person | Vi_y | Yes | 2 | 4% | 2 | 5% | **5** | **71%** | 9 | 9% |
|  |  | Vi_n | No | 54 | 96% | 37 | 95% | 2 | 29% | 93 | 91% |
|  | Isolating oneself from others with whom one lives | Is_y | Yes | 1 | 2% | 0 | 0% | **7** | **100%** | 8 | 8% |
|  |  | Is_n | No | 55 | 98% | 39 | 100% | 0 | 0% | 94 | 92% |
|  | Having a “telehealth visit” with a doctor or other health care provider | Te_y | Yes | 1 | 2% | 6 | 15% | 1 | 14% | 8 | 8% |
|  |  | Te_n | No | 55 | 98% | 33 | 85% | 6 | 86% | 94 | 92% |
|  | Working or studying at home | Ho_y | Yes | 0 | 0% | 2 | 5% | 3 | 43% | 5 | 5% |
|  |  | Ho_n | No | 56 | 100% | 37 | 95% | 4 | 57% | 97 | 95% |
|  | Cancelling/postponing work or school activities | Ca_y | Yes | 0 | 0% | 2 | 5% | 2 | 29% | 4 | 4% |
|  |  | Ca_n | No | 56 | 100% | 37 | 95% | 5 | 71% | 98 | 96% |

Shaded cells indicate the most frequent answers in the same cluster.

The shaded numbers indicate an opposite trend from the overall answer.
