# Supplementary material for: Exploring ways to support patients with noncommunicable diseases: A pilot study in Nepal during the COVID-19 pandemic
Source: PLOS Glob Public Health. 2024 Jul 19;4(7):e0003509. doi: 10.1371/journal.pgph.0003509 (PMC11259295; doi:10.1371/journal.pgph.0003509)
Supplement: Attachment — Submitted filename: comment_20240322.docx [file pgph.0003509.s005.docx]

**Response to Reviewers**

***Reviewer #1: Overall evaluation:
This is an interesting pilot study investigating the experiences and challenges faced by patients with noncommunicable diseases (NCDs) in Nepal during the COVID-19 pandemic. The study uses a mixed-methods approach combining both quantitative survey data and qualitative interviews to explore healthcare service availability and barriers to care for NCD patients.***

***The importance of the research question is clear - NCD patients are at higher risk for poor COVID-19 outcomes,*** ***yet little is known about how the pandemic impacted their healthcare access and disease management, especially in low-resource settings like Nepal. The objectives are specific, and the mixed-methods approach is appropriate. The sample sizes seem reasonable for an initial pilot study. The analyses utilize appropriate statistical tests and qualitative methods. The discussion situates the findings well within the larger literature and considers implications and future directions.***

***Overall, this manuscript makes a meaningful contribution to understanding NCD care amidst the COVID pandemic. I recommend minor revisions to strengthen the presentation and highlight key results prior to acceptance for publication.***

【Authors】

Thank you very much for your important comments. We have responded to you as follows.

***Major comments:***

***1. The abstract and introduction could more clearly distinguish the study's novel contributions from what is already known about COVID's impacts on global NCD care per WHO reports.***

【Authors】

The followings were added in Introduction.

Line 85, “*Although the WHO has already noted the importance of health systems in the fight against NCDs [15], further knowledge is needed to ensure that these systems are even stronger when emergencies such as the COVID-19 pandemic occur.*”

Unfortunately, the abstract could not include explanations due to the word limit, so we have tried to enrich it in Introduction.

***2. Consider restructuring parts of the results section for clarity and concision:***

- ***Combine results for Objectives 1 and 2 which both rely on the qualitative data***

***- Trim the MCA, HCA, and cluster details that do not directly address the research questions (perhaps move some details to supplement)***

***- Prioritize highlighting key survey results on healthcare access challenges***

【Authors】

Yes. We changed not only the Results section, but also the structure of the last paragraph of Introduction, Materials and Method.

Introduction concludes with the following sentences without detailing the objectives.

Line 106, “*Therefore, this study aimed to understand the experiences of and challenges faced by patients with NCDs in Nepal during the pandemic, focusing on healthcare service availability and identifying factors affecting healthcare use.*”

Materials and Methods was divided into new paragraphs with the following subheadings.

“Study setting”→ “Study design”→ “Study population”→ “Sampling technique”→ “Data collection”→ “Ethics approval”→ “Data analysis”

The explanation of MCA and HCA in Materials and Methods has been moved to the S2_Text.

Results section was divided into paragraphs with the following subheadings.

“Baseline information” → “Situation of patients with NCDs during the COVID-19 pandemic” → “Potential barriers for service utilization” → “Facilitators of the use of healthcare” → “Useful field information obtained through exploratory methods”

The following text in the last paragraph of Result section was moved to Discussion.

Line 376, “*Furthermore, MCA and HCA methodology allowed us to identify an ideal patient population (Cluster 3) in which proper countermeasures appeared to have been undertaken (S1 Table). We found that MCA and HCA were useful for quickly finding relationships and structures with limited data from telephone interviews in situations where face-to-face contact is avoided. A more in-depth, qualitative investigation of this Cluster 3 would have uncovered suggestive behaviors, however, this was unfortunately not possible because direct interviews and observations were avoided.*”

The order of questions in Table 1 was also changed.

***3. The discussion could comment further on:
- why access barriers existed when services were reportedly still available***

【Authors】

The following explanation was added to explain that access is a different situation for each patient.

Line 382, “*Although the qualitative approach indicated that health care and drug provision services were barely normal during the study period, we found that each patient had their own individual problems. For example, even if medicine could be procured, if no doctor was available, the appropriate prescription for the medicine could not be determined.*”

***- the finding that only 1 participant used telehealth***

【Authors】

The following was added in Discussion.

Line 398, “*In this study, only one of the patients who canceled their visit with their doctor had access to telemedicine; hopefully, in the future, all patients will have this option.*”

***- comparisons to NCD impacts observed in other low- and middle-income countries***

【Authors】

NCD impact occurs similarly in all countries, and instead of making comparisons, we explained as follows.

Line 422, “*Globally, when considering both low-and high-income countries, the COVID-19 pandemic caused a general decline in health service use and exposed a variety of barriers to service use, even for routine treatment of NCD [30]*.”

***4. Address generalizability limitations: convenience sampling from only 2 districts***

【Authors】

We added the followings in “Limitation”.

Line 477, “*First, as convenience sampling from only two districts was used, there may be concerns regarding the generalizability of the findings.*”

***Minor comments:***

***Abstract***

***1. Outline the specific methods in brief (quant survey and qualitative interviews)***

【Authors】

We added the followings.

Line 39, “*We used mixed methods, with both qualitative and quantitative approaches. Specifically, multiple correspondence analysis, hierarchical cluster analysis, and classification tree analysis were used as exploratory methods.*”

***2. The conclusion could more directly connect back to the study objectives and purpose***

【Authors】

Thank you very much for your comment. We have made that change.

Line 48, “*Our findings suggest the need to design and implement telemedicine services for patients with NCDs, as well as community-based programs that aim to improve health literacy, encourage healthy behavior, prevent development of NCDs, and ensure continuity of care during such crises, especially in countries with limited resources*.”

***Introduction***

***1. References 6 and 14 seem potentially irrelevant for context specific to Nepal - consider replacing with local prevalence/burden data if appropriate***

【Authors】

About Reference 6, we erased the following sentence.

~~“A meta-analysis by Yang et al. (2020) revealed that the most prevalent comorbidities, in the context of COVID-19, were hypertension (21.1%) and diabetes (9.7%), followed by cardiovascular (8.4%) and respiratory system (1.5%) diseases [6].”~~

Instead, we added the following sentence.

Line 61, “*According to a cross-sectional study conducted by Amatya et al. in Nepal between 2020 and 2021, the most commonly present comorbidity was hypertension (43.7%), followed by diabetes (25.8%) [6]*.”

About Reference 14, this reference was retained here, with the following sentence and a new citation added.

Line 79, “*The same is true in Nepal, especially among older adults with pre-existing noncommunicable conditions [16]*.”

***Methods***

***1. Were interviews conducted with any of the same patients included in the quantitative sample? Clarify whether qualitative and quant participants overlapped.***

【Authors】

The following was added.

Line 144, “*Regarding patients, there was overlap in the interviewees for the qualitative and quantitative approaches, while regarding non-patients, the interviewees were addressed using the qualitative approach only.*”

***2. Verify whether verbal or written consent was obtained (text seems contradictory)***

【Authors】

Thank you very much for your precise comment. We changed to the followings.

Line 120, “*Owing to travel restrictions, interviews with the participants were conducted via telephone*”, because we have already mentioned as below.

Line 160, “Verbal consent was obtained from each participant via telephone.”

***Results***

***1. Consider organizing the qualitative results by the key themes in barriers/facilitators***

【Authors】

Yes. We considered as your comment. First, we put subheading instead of Objective 1, 2, 3, according to the other reviewer’s comment as “***11) Please provide meaningful subheadings instead of Objective 1, 2, etc.”*** According to new subheadings, the results were arranged including qualitative results. Many patterns were tried considering your comment, and we decided current version. Thank you very much for your comment.

***2. For Fig 1: define "variables” analyzed in MCA plot (questions from survey?)***

【Authors】

The following description was added to the legend in Figure 1.

“*The variable names are as shown on the right side of Table 1*.”

***Discussion***

***1. In limitations: Note social desirability bias for phone interviews specifically***

【Authors】

The followings were added in limitation part.

Line 487,“*Fourth, we could not control for social-desirability bias, which may have led the participants to provide answers that would make them look good to the researcher [44]. Holbrook et al. indicated that social desirability bias might occur more frequently in telephone interviews compared to face-to-face interviews [45]. On the other hand, because it was a telephone survey, participants were able to answer more honestly, in some cases [46], especially in emergencies, such as the COVID-19 pandemic. In face-to-face interviews, eye contact may be intimidating for some participants [47]*.”

***Tables/Figures - Well designed to support key results***
***References - Comprehensive and adequate***
***In summary, I recommend minor revision to better highlight the key findings before final acceptance. This pilot study makes a valuable contribution towards understanding NCD care access issues amidst COVID-19 in Nepal's resource-limited setting. Addressing the clarity/concision of results and highlighting novel contributions will further strengthen the manuscript for publication. Please feel free to contact me if you have any other questions!***

【Authors】 Thank you very much for your input.

***Reviewer #2: The study involved collecting questionnaire responses from NCD patients pertaining to their situation and concerns during COVID-19. The Authors devised an approach using MCA and HCA as well as qualitative analysis. The study identified many important situations that demands attention for remedies in the event of next pandemic. The results from the questionnaire is found to be insightful and the paper presents useful measures for pandemic preparedness. However, in its present form, it cannot be accepted due to the weakness found in using MCA. Furthermore, it requires language editing and clarifications as follows.***

【Authors】 Thank you very much for your comment. The study design is not perfect in some respects because we needed to conduct the study as quickly as possible in the emergency situation, but we have tried to present the data as accurately as possible.

***1) Line 86, did reference 15 report on the challenges and behavior of the people? Include relevant literature that also performed similar studies. Can references of similar studies of conditions faced by people with or without NCD during COVID-19 be provided? The paper is lacking of literature review of studies of understanding the situation of patience with illnessess or NCD during COVID-19.***

【Authors】

Thank you very much for your comment. We checked again because a lot of time had passed since the first draft. The following was added.

Line 79, “*The same is true in Nepal, especially among older adults with pre-existing noncommunicable conditions [16].*”

***2) Line 110, objective 1, why is there a special mention of diabetes? Is there a particular focus on diabetes and why? Please clarify.***

【Authors】

“Line 119” is correct? Is so, “*diabetes and other*” should have been erased. Finally, the description of the detailed Objectives was changed, as other reviewers commented on it. We used the “patients with NCDs” as below.

Line 106, “*Therefore, this study aimed to understand the experiences of and challenges faced by patients with NCDs in Nepal during the pandemic, focusing on healthcare service availability and identifying factors affecting healthcare use.*”

***3) Line 121, Clarify objective 2, "to the use of diabetes", what do you mean by that?***

【Authors】

The sentence should have been changed as below.

“the use of services for people with NCDs”. Finally, the content of Objective 2 is included in the above sentence describing the aim of this study.

***4) Line 139, remove the word purposively.***

【Authors】 Yes. Thank you very much.

***5) Line 167 - 199, it was not mentioned why these methods of analysis were chosen and in what way will these methods satisfy the aim of the study. There are many other methods which could also do the same analysis.***

【Authors】

Thank you very much for your comment. This is an exploratory study, and a variety of methods could be adapted, but a detailed explanation is provided in answer for **“*15) How can you verify that the MCA and HCA results are the correct or best ones to present?”***

The explanation “*as exploratory method*” is added in the first sentence in “Data analysis” of Method part. (Line 162)

***6) What were similar studies that used the approaches used in this paper? Why are these approaches considered suitable? Please explain.***

【Authors】 This study is a pilot study and there would be no studies using a similar survey methodology. Even in the emergency situation of the COVID-19 pandemic, we sought means to share information as scientifically as possible using our own methods. We first checked the characteristics of the data, then used a variety of techniques, and finally settled on this method as the best way to describe the data.

***7) Table 1, Provide number references to the questions in Table 1 for easy reference including the relevant questions reference numbers in the qualitative part of the study.***

【Authors】 Order of the questions in Table 1 are changed and displayed from A-1 to E-5. The number of each question was also added to the explanation of Table 1 in the text.

***8) What does the * mean in Table 1 in MCA column?***

【Authors】 Instead of “Not applicable”, each explanation was included in each cell of Table 1.
A-2. Gender: “Others” are removed.

B-1. Are you currently taking medicines for chronic conditions? →“Not used in MCA” are added.

***9) What does it signify when the no. of patients in MCA is different from actual? Is there a bias in MCA towards categories with more samples e.g in Religion?***

【Authors】 Because the MCA uses binary variables, for those with more than one answer choice, the answers should be divided into binary values to take into account the nature of the answers for the MCA analysis. Baseline information, such as religion, was divided into binary variables in the MCA and HCA, but most baseline information was not an important variable for clustering in the HCA. Even though the baseline information was treated as a more detailed categorical variable rather than a binary variable, this study did not believe it reduced the credibility of the results. Moreover, as an implication of this study, we also consider the possibility that people's behavior is determined without regard to the religion of this study or any other.

***10) Line 252, The cummulative variance that indicates how much of the data is explained in the plot is less than 30%. Please show the variance and cummulative variance of each dimension. How can you justify or verify that what is reported regarding the contributing features to the clusters is true? This indicate that MCA biplot is not reliable and is not suitable analysis for to study the data.***

【Authors】 Percentage of explained variances of our data is as below.

While it is clear that low cumulative variance in MCA can be a concern, there is no universally defined threshold for how much cumulative variance is acceptable; it is important to carefully consider the specific context and characteristics of the data when interpreting cumulative variance in MCA, and this study uses a mixed method to compensate for its low cumulative variance.

***11) Please provide meaningful subheadings instead of Objective 1, 2, etc.***

【Authors】

Thank you very much for your comment.

Rather than detailing objectives 1,2 and 3 at the end of Introduction, the aim was described in one sentence as blow.

Line 106, “*Therefore, this study aimed to understand the experiences of and challenges faced by patients with NCDs in Nepal during the pandemic, focusing on healthcare service availability and identifying factors affecting healthcare use.*”

Accordingly, the subheading in Results is also as follows.

“Baseline information” → “Situation of patients with NCDs during the COVID-19 pandemic” → “Potential barriers for service utilization” → “Facilitators of the use of healthcare” → “Useful field information obtained through exploratory methods”

***12) Line 253, Please show the dendogram for the HCA so that we can see the hierarchy of the clustering and the cut-off where the 4 clusters are found. It is unclear why 4 groups are most suitable.***

【Authors】 Thank you very much for your important comment. The following two dendrograms were checked again and changed, assuming that three clustering were optimal. The descriptions of Cluster 4 in the draft have been deleted.

***13) Please align the respective objectives with the questionnaire questions listed in Table 1 so that we can see the prevalence of that response.***

***E.g People's fear of getting infected by health workers was drawn from the question avoided in-person contact with high risk people or its question reference number.***

***Or if such objectives can be included in Table 1, for us to easily understand the prevalence of such behaviors***

【Authors】

Thank you very much for your comment. Other reviewers have commented on this point, and we have considered various patterns. The order of variables in Table 1 was changed and their numbers were clearly indicated.

In particular, our mixed method followed the convergent design. We collected both qualitative and quantitative data concurrently, analyze them separately, and then merge or compare the results to gain insights into the research topic.

In each question in Table 1 is numbered so that the corresponding question can be identified in the text in Results section.

***14) Can the study of objectives sections be more structured and objective? How many patients reported fear of getting infected, as well as the other potential barriers and what are the demographics of such patients? How many are from Kavrepalanchok and Nuwakot? How many facing which barriers are having which NCD?***

【Authors】

Thank you very much for your comment. Various patterns of changes were considered, taking into account the comments of other reviewers.

As we have mentioned above, we have removed the detailed statement of objectives (1,2, and 3) and left the aim only in last part of Introduction.

This study used exploratory methods, including CTA and MCA, and considered that only characteristic results should be described. For example, a characteristic result of CTA is shown in Figure 2. The text in “Useful field information obtained through exploratory methods” (Line 340) is the characteristic result. There was nothing of note regarding baseline information, such as “what are the demographics of such patients”.

Regarding the region, Kavrepalanchok and Nuwakot were treated as regions with similar characteristics and were not distinguished. Although regional differences could not be taken into account, differences in baseline information (Table 1, A) were considered important for the analysis. In our exploratory approach, only the results described in this draft were characteristic results and there were no characteristic results related to baseline information other than regional differences.

For just in case, the fact that regional differences were not taken into account is noted in Limitation as follows.

Line 479, “*In addition, differences between the two regions (Kavrepalanchok and Nuwakot) were not taken into account in the analysis*.”

***It is unclear the benefits of showing the transcripts of the recordings. Please justify the benefits in the introduction as well as elaborate on the reason for presenting only the recordings and not other statistical analysis of the survey with regards to this objective, apart from the clustering.***

【Authors】

Instead of explaining the method in Introduction, the structure of the Method was changed to make the whole process of data analysis easier to understand. The subheadings were also added to the Results, including recordings, to correspond with the Method.

Some transcripts of the recordings were erased, such as below.

“*I like this concept of telemedicine. It will be very effective.*” (Th001, Patient, Diabetes and Thyroid disorder)”

***15) How can you verify that the MCA and HCA results are the correct or best ones to present?***

【Authors】 This study is an exploratory study in an emergency situation. If there had been a reliable outcome setting, for example COVID-19 negative or positive, would have provided a clear indication of the analysis method. However, at the time this study was conducted, no testing system was available, making it difficult to establish outcomes. Therefore, we recognize that the MCA and HCA results were the best available.

***16) Line 382, while the Authors claim that data mining methods were used to obtain as much as possible, however the recordings presented in the objective sections can be mined for further analysis.***

【Authors】

Thank you very much for your comment. We thought that our use of the term "data mining" was inappropriate. The term “data mining methods” in this draft has been changed to “exploratory methods”.

In this study, we tried to report as much as possible on facts that we were able to discover in an exploratory manner, even if we were unable to design a hypothesis. In such a case, we thought "exploratory methods" would be a better expression.

The sentence was changed to

Line 341, “*In this study, exploratory methods were used to obtain information about the situation in the field during the COVID-19 pandemic.*”

***17) Line 471, the negative feelings evoked by social media were stronger in patients who were concerned about COVID-19 and ...... than who? Please specify, it is unclear what is being compared here.***

【Authors】

The followings were added.

Line 437, “*This study showed that negative feelings evoked by social media were common in patients who were concerned about COVID-19 and encountered difficulty in obtaining their medicine (Figure 2, "Nervous by social media" response is more common in Box C than in Box B)*.”

***18) Line 491, please rephrase - it is unclear what is meant by "secrets of health behaviour" and also what is the "Nepalese way", please explain to make it understandable to non-Nepalese readers***

【Authors】 We changed to as below.

Line 457, “*This approach involves identifying contextual health behaviors in Nepal, which may be disguised as “deviant behavior,” and disseminating them to the local community.*”

***Reviewer #3: The sentence structure of most sentences in the manuscript needs improvement because they are difficult to understand.***

【Authors】 English proofreading has already been outsourced to a company.

***The methodology should be presented more systematically and simply, with details such as the study population, sampling method, data collection procedures, and analysis methods used for both qualitative and quantitative studies. It is also important to follow a standard format for tables and diagrams.***

【Authors】 Thank you very much for your comment. This is an exploratory and pilot study and we struggled with how to clearly display the methods. Finally, the structure of the Method was changed to make the whole process of data analysis easier to understand.

***Table 1 is too lengthy and needs to be simplified to highlight important findings. Other results also need to be simplified to highlight the important findings. Additionally, the term "telemedicine" needs to be defined.***

【Authors】

As answer to ***“7) Table 1, Provide number references to the questions in Table 1 for easy reference including the relevant questions reference numbers in the qualitative part of the study.”****,* the questions in Table 1 are displayed from A-1 to E-5. The number of each question was also added to the explanation of Table 1 in the text. The subheading of the results was adjusted.

The definition of telehealth/telemedicine here is consultation with doctors and nurses through the internet or by phone. This was mentioned in S1 Text: Interview guide for patients with type 2 diabetes. In Discussion, we stated the following.

Line 404, “*Telemedicine, defined as the provision of healthcare services remotely through the utilization of digital platforms and technology by healthcare professionals [24], manifested as a promising solution during the COVID-19 pandemic.*”

***Furthermore, there is a discrepancy where the total percentage of participants with no difficulty accessing routine medical care and the percentage of participants with challenges in accessing routine medical care add up to more than 100%.***

【Authors】 Perhaps readers may find the following sentences difficult to understand, so we have separated the sentences in Abstract.

Line 41, “*The study revealed that while 69.6% of the participants reported no difficulty in obtaining medication, other questions revealed that 58.5% experienced challenges in accessing routine medical care*.”

***Solutions were proposed for two barriers: telemedicine and community-based programs were suggested for addressing fears of infection and lack of transportation. However, no solution was proposed for the unavailability of medicine.***

【Authors】

Your point is quite correct. As you noted, we tried to add patient opinions regarding a solution to the lack of access to medicines. In this study, opinions on “Free medicine” were raised. However, we rescinded that statement because this opinion had been discussed before the COVID-19 pandemic.

Since this study was a pilot study and was not perfect, we would like to create a research design in future studies that can properly respond to the all comments we received.

Thank you very much for your valuable comments.
